# Diseases Admitted as in and Out-Patients under the Care of the Physicians of the Westminster Hospital, from the 20th of July to the 20th of August

**Published:** 1799-09

**Authors:** 


*Diseases admitted as In and Out-Patients under the care of the Physicians of the Westminster Hospital, from the 20th of July to the 20th of August.*

| | |
|---|---|
| Fevers - - - | 14 |
| Hepatitis - - | 1 |
| Quotidian - - - | 1 |
| Tetanus - - | 1 |
| Amenorrhœa - - | 4 |
| Anasarca - - - | 2 |
| Ascites - - - | 4 |
| Epilepsy - - | 1 |
| Gastrodynia - - | 2 |
| Hooping Cough - | 1 |
| Hemoptoe - - | 2 |

| | |
|---|---|
| Asthenia - - - | 6 |
| Asthma - - - | 1 |
| Cough - - - | 4 |
| Diarrhœa - - | 9 |
| Dyspepsia - - - | 3 |
| Dysuria - - | 1 |
| Enterodynia - - | 2 |
| Hemorrhois - - | 1 |
| Hypochondriasis - | 3 |
| Impetigo - - | 1 |

Itch

| | | | |
|---|---|---|---|
| Itch | 2 | Pleurisy | 1 |
| Jaundice | 1 | Rheumatism | 9 |
| Lepra Græcorum | 1 | Sciatica | 1 |
| Lumbago | 2 | Struma | 4 |
| Menorrhagia | 2 | Urticaria | 1 |
| Phthisis | 1 | Tinea | 2 |
| Paralysis | 3 | Worms | 3 |

